# Indoleamine 2,3-Dioxygenase 1 (IDO1) in Kidney Transplantation: A Guardian against Rejection

**DOI:** 10.3390/jcm12247531

**Published:** 2023-12-06

**Authors:** Krzysztof Wiśnicki, Piotr Donizy, Agnieszka Hałoń, Patryk Wawrzonkowski, Dariusz Janczak, Magdalena Krajewska, Mirosław Banasik

**Affiliations:** 1 Department of Nephrology and Transplantation Medicine, Wroclaw Medical University, 50-367 Wroclaw, Poland; p.wawrzon@gmail.com (P.W.); magdalena.krajewska@umw.edu.pl (M.K.); 2 Department of Clinical and Experimental Pathology, Wroclaw Medical University, 50-367 Wroclaw, Poland; piotr.donizy@umw.edu.pl (P.D.); agnieszka.halon@umw.edu.pl (A.H.); 3 Department of Vascular, General and Transplantation Surgery, Wroclaw Medical University, 50-367 Wroclaw, Poland; dariusz.janczak@umw.edu.pl

**Keywords:** kidney transplantation, acute rejection, indoleamine 2,3-dioxygenase 1 (IDO1), immunosuppression, antibody-mediated rejection (AMR), T-cell mediated rejection (TCMR)

## Abstract

Kidney transplantation is a crucial treatment for end-stage kidney disease, with immunosuppressive drugs helping to reduce acute rejection rates. However, kidney graft longevity remains a concern. This study explores the role of indoleamine 2,3-dioxygenase 1 (IDO1) in kidney transplant immunology. IDO1 breaks down tryptophan, affecting immune cell behavior, primarily T-cells. The research focuses on both cellular and antibody-mediated immune responses, often causing graft damage. The study assessed IDO1 expression in renal transplant biopsies from patients with graft function decline, examining its connection to clinical parameters. A total of 121 biopsy samples were evaluated for IDO1 expression using immunohistochemistry. Patients were categorized as IDO1(+) positive or IDO1(−) negative based on immunoreactivity in tubular epithelium. Results showed a significant link between IDO1 expression and rejection incidence. IDO1(+) positive patients had lower rejection rates (32.9%) compared to IDO1(−) negative ones (62.2%) [*p* = 0.0017], with substantial differences in antibody-mediated rejection (AMR) (5.2% vs. 20%) [*p* = 0.0085] and T-cell mediated rejection (TCMR) (31.6% vs. 57.8%). These associations suggest that IDO1 may play a protective role in kidney transplant rejection. IDO1 modulation could offer novel therapeutic avenues to enhance graft survival. The study underscores IDO1 as a potential marker for rejection risk assessment, with its potential applications in personalized interventions and improved patient outcomes. Further research is needed to fully comprehend the mechanisms behind IDO1’s immunomodulatory functions and its potential clinical translation.

## 1. Introduction

Kidney transplantation is the most effective treatment for patients with end-stage kidney disease, significantly enhancing their lifespan and quality of life [1,2,3,4]. Immunosuppressive drugs, particularly those targeting T-cell function, have lowered the incidence of acute rejection [5,6]. However, the longevity of transplanted kidneys remains a concern [7,8,9].

Acute kidney graft rejection is a severe complication that can occur after a kidney transplant [10,11]. Patients who experience this condition require prompt medical attention to prevent further damage to the transplanted organ. The standard treatment for acute kidney graft rejection typically involves a combination of immunosuppressive medications, such as corticosteroids and anti-rejection drugs [12].

In a review that we have published [13], we have summarized the mechanisms associated with IDO1 and outlined its significance in the immunological response of kidney graft recipients [14]. Indoleamine 2,3-dioxygenase (IDO1) is an intracellular enzyme that breaks down tryptophan to produce kynurenine metabolites [15,16,17]. This leads to reduced levels of tryptophan and increased levels of its metabolites, causing cell-cycle arrest and inducing apoptosis of effector T-cells while promoting the activity of regulatory T-cells [18,19,20,21]. IDO1 acts on most immune cells, including monocytes, macrophages, and dendritic cells [22,23,24], but it primarily affects lymphocytes [25]. The precise role of IDO1 in regulating local immune balance in kidney transplant patients is not yet fully understood. Up to this point, researchers have primarily focused on the role of IDO1 in the cellular immune response, particularly concerning T-cells, their proliferation, and behavior [26,27,28,29,30].

Nonetheless, it is essential not to overlook the humoral, antibody-based immune reaction concerning IDO1. As mentioned earlier, it becomes imperative to explore novel methods of safeguarding transplanted organs. The primary contributor to graft immunological damage and transplant failure is antibody-mediated rejection, with the human leukocyte antigen (HLA) and the presence of anti-HLA and non-HLA antibodies playing a significant role [10,31,32,33,34,35].

Our study aims to assess the expression of IDO1 in renal transplant biopsies obtained from patients experiencing a decline in graft function and to explore the relationship between IDO1 expression and various clinical parameters. Patients with kidney function deterioration, some of whom have proteinuria, routinely have a graft biopsy performed to assess the injuries and provide proper therapy [36,37]. Then, ordinarily, a standard treatment of graft rejection is introduced [12,38]. While intravenous steroids and T-cell depletion are the first-line treatment in T-cell-mediated rejection [39], plasmapheresis and intravenous immunoglobulins cope with the antibody-mediated rejection [12].

## 2. Materials and Methods

### 2.1. Collecting Patients and Samples

The research involved 121 patients who received renal transplantation, had a progressive graft dysfunction documented, and were hospitalized between August 2011 and June 2016 in the Clinical Department of Nephrology and Transplantation Medicine of the University Clinical Hospital in Wroclaw. Due to renal function deterioration, as a standard of clinical care, graft biopsies were performed.

All of the biopsies were conducted because of progressive graft dysfunction, which we understand as an increase of serum creatinine level by 0.3 mg/dL or more. Serum creatinine was routinely checked every three months in the outpatient clinic. In the event of an increase in creatinine, we decided to perform a biopsy.

All rejection episodes were indeed detected in the same biopsy in which IDO1 expression was analyzed. This direct correlation between IDO1 expression and rejection events in the same biopsy enhances the specificity of the association and underscores the temporal relationship between IDO1 expression and rejection.

Among the 121 patients, 74 had proteinuria as an additional reason to perform the biopsy. Intentionally, all of the biopsies were performed before any kind of additional immunosuppressive treatment was administered; thus, the expression of IDO1 would not be altered.

In our scientific study, it is essential to highlight that none of the patients involved were subjected to additional risk due to our investigation. Given the inherent necessity for kidney transplant recipients to undergo biopsies as a result of deteriorating graft function, our study merely aligned with the established medical protocols, ensuring that the patients were not exposed to any additional hazards beyond the standard procedures associated with the evaluation of transplant outcomes. Importantly, written, informed consent was gathered from all participants. The patients came exclusively from the aforementioned single medical center. The research protocol was approved by the Research Ethics Board of Wroclaw Medical University (KB 628/2021). This underscores the ethical considerations and precautionary measures taken to safeguard the well-being of the participants throughout the course of our research.

### 2.2. Histopathology and Staining Method

In our study, a retrospective analysis of 121 renal transplant biopsies was performed, and the immunohistochemical expression of IDO1 in tubular epithelium (TE) was assessed. The scores for TE were dichotomized into two categories: no expression or any expression. Immunohistochemistry analyses were conducted on 4-micrometer-thick sections of tissue using primary antibodies directed against IDO1 (1F8.2, 1:400, Millipore, Burlington, MA, USA) [40,41]. To retrieve the epitope, heat was applied to the slides using EnVision Target Retrieval Solution (Agilent DAKO, Santa Clara, CA, USA) [42] in a 30-min incubation at 97 °C in PT Link Pre-Treatment Module for Tissue Specimens (Agilent DAKO, Santa Clara, CA, USA) [43]. The automated immunohistochemical staining was carried out using Autostainer Link 48 (Agilent DAKO, Santa Clara, CA, USA) [44], and the detection system utilized was Liquid Permanent Red (Agilent DAKO, Santa Clara, CA, USA) [45].

**Figure 1:**
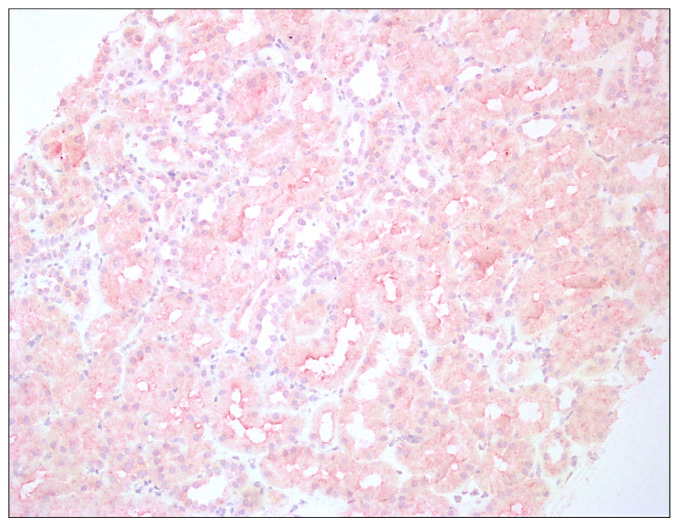
Enhanced immunoreactivity of IDO1 in tubular epithelium of renal graft; hematoxylin, 200×.

**Figure 2:**
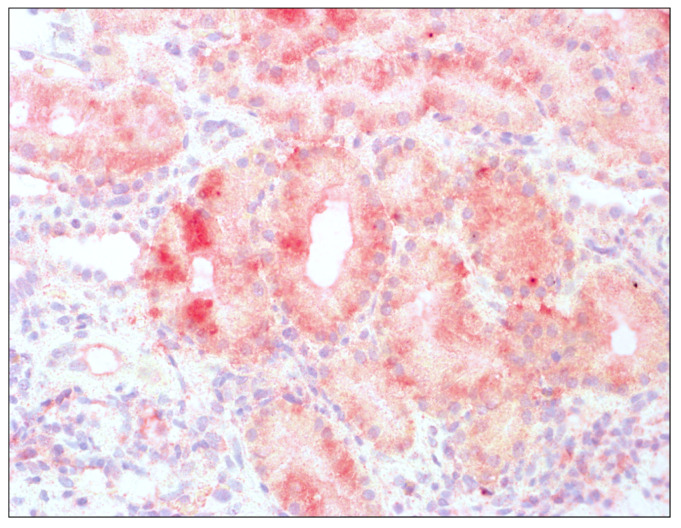
Enhanced immunoreactivity of IDO1 in tubular epithelium of renal graft; hematoxylin, 400×.

Histopathological assessment of transplant biopsies is presented in Figure 1 and Figure 2. The captions contain information about methods of staining and magnification used.

### 2.3. The Scoring System for IDO1 Expression

In our study, the assessment of immunoreactivity in tubular epithelium (TE) was conducted, employing a three-point scale to capture the nuanced variations in expression. The scale ranged from 0, denoting an absence of immunoreactivity, to 1, indicating a subtle or slight expression, and 2, signifying a more pronounced and moderate to high immunoreactivity.

To further elucidate the immunohistochemical findings, the TE scores were categorized into two distinct groups: negative and positive. The IDO1(−) negative group, comprised 45 patients who exhibited no discernible IDO1 expression, registering a score of 0. Conversely, the IDO(+) positive group, encompassed 76 patients characterized by any degree of IDO1 expression, scoring either 1 or 2 on the scale.

This classification strategy not only provided a concise representation of the immunoreactivity levels within the tubular epithelium but also facilitated a clear demarcation between cases with and without IDO1 expression. The IDO1(−) negative and IDO1(+) positive groups, thus formed, set the stage for a comprehensive analysis of the implications of IDO1 expression in the context of our study population, fostering a more detailed understanding of the observed patterns and associations.

### 2.4. Sensitivity and Specificity of the Study

In the context of analyzing the properties of the IDO1 marker as a predictor of transplant rejection, it is important to consider the statistical metrics for both sensitivity and specificity.

In this particular study, a challenge arises from intuitively assigning a positive logical value to an event that is negative in nature (transplant rejection). This inherent difficulty in the logical assignment can complicate both the design of the analysis and subsequent interpretation. To address this issue, the Classification and Regression Training package in R version 4.3.2 (R Foundation for Statistical Computing, Vienna, Austria) was employed, acknowledging the complexities of the analysis despite the seemingly straightforward formulas.

Sensitivity, denoted as 0.472 in this study, signifies the test’s ability to correctly identify individuals with a lower risk of rejection based on IDO1 expression. A sensitivity of 0.472 implies that the test accurately identified 47.2% of patients with lower rejection rates, indicating a moderate ability to capture true positives. Conversely, specificity, indicated as 0.250, denotes the test’s accuracy in identifying individuals at a higher risk of rejection due to the absence of IDO1 expression. A specificity of 0.250 suggests that the test correctly identified 25% of patients with higher rejection rates.

While the specificity is relatively low, augmenting the study with a larger number of patients would possibly contribute to enhancing its clinical reliability in identifying patients at varying risk levels of rejection based on IDO1 expression.

### 2.5. Statistical Tools and Software Used for Data Analysis

Statistica version 14.1.0 (TIBCO Software, Warsaw, Poland) and R version 4.3.2 (R Foundation for Statistical Computing, Vienna, Austria) were used for statistical analysis. A *p* value below 0.05 was considered significant. A Student *t*-test, Mann–Whitney U test, and chi-squared test were applied for statistical analysis. The dataset, comprising information on the presence of IDO1 in the tubular epithelium of kidneys from 121 kidney transplant patients, was imported into the respective platforms.

### 2.6. Proteinuria as an Additional Indication for Biopsy

As mentioned above, among the 121 patients under consideration, 74 individuals presented with proteinuria as an adjunctive factor necessitating biopsy. Within the subset of patients testing positive for IDO1, comprising 76 individuals, 44 of them (57.89%) exhibited proteinuria. Conversely, in the IDO1(−) negative group encompassing 45 patients, 30 individuals (66.67%) met the criterion of proteinuria. Notably, statistical analysis did not reveal a significant difference between these two groups, as evidenced by a *p* value of 0.157.

### 2.7. Time Elapsed between Transplantation and Biopsy

In examining the temporal dynamics between transplantation and biopsy within the entire cohort of patients, the data revealed a median duration of 163 weeks. To delve deeper into this time frame, a distinct divergence emerged between the IDO1(−) negative group and the IDO1(+) positive group. Specifically, the median interval in the IDO1(−) negative cohort stood at 43 weeks, underscoring a comparatively swifter timeline. In contrast, patients in the IDO1(+) positive group exhibited a more extended median duration of 203 weeks.

This temporal distinction between the two groups yielded a statistically significant finding, as denoted by a *p* value of 0.01928.

## 3. Results

### 3.1. Characteristics of Patients

The most important characteristics of patients are shown in Table 1.

In terms of immunosuppressive therapy, all of the patients were receiving calcineurin inhibitors; in 85 cases, they received tacrolimus and, in 36 cases, cyclosporin. Additionally, all of them received mycophenolate mofetil. In addition to that, two received azathioprine, and two received an anti-CD25 therapy [12,46,47].

The initial immunosuppression is shown in Table 2.

In the gathered 121 patients, the most common cause of native kidney injury was chronic glomerulonephritis (51 cases), diabetic nephropathy (24 cases), followed by hypertonic nephropathy and polycystic kidney disease (11 cases each).

The causes of chronic renal failure are presented in Table 3.

The results have been summarized in Table 4.

### 3.2. Rejection in Patients with Expression of IDO1

Interestingly, among the 76 patients with IDO1 expression in tubules, only 25 individuals experienced rejection episodes, accounting for a rejection incidence rate of 32.9%. In contrast, within the group of 45 patients lacking IDO1 expression in tubules, a higher proportion of 28 individuals faced rejection, resulting in a rejection incidence rate of 62.2%. The stark contrast in rejection rates between the two groups prompted a closer examination of the impact of IDO1 on rejection outcomes. The observed difference in rejection incidence proved to be statistically significant, with a *p* value of 0.0017.

### 3.3. Analysis of the Occurrence of Antibody-Mediated Rejection

A noteworthy finding from our analysis showed that antibody-mediated rejection (AMR) occurred at a significantly lower rate in patients with IDO1 expression, with only 5.2% of IDO1(+) positive patients experiencing AMR episodes. In contrast, AMR was observed in 20% of IDO1(−) negative patients, a substantial difference that emerged as statistically significant (*p* = 0.0085).

### 3.4. T-Cell Mediated Rejection Manifestation

Our study further delved into the intricate interplay between IDO1 expression and T-cell mediated rejection (TCMR) in kidney transplant recipients. Through an analysis of biopsy samples from patients experiencing both pure and mixed rejection, we made noteworthy observations regarding the impact of IDO1 on TCMR incidence. Interestingly, in both groups, TCMR was detected. Its occurrence, however, was significantly lower in the IDO1(+) positive class, representing only 31.6% of cases. In contrast, the IDO1(−) negative group exhibited a higher TCMR prevalence, accounting for 57.8% of cases. Notably, these differences proved to be statistically significant (*p* = 0.0046), underscoring the potential role of IDO1 in tempering T-cell-mediated rejection events.

### 3.5. Pure Types of Rejection

In our investigation, we found that the incidence of pure TCMR did not demonstrate a statistically significant difference between IDO1(+) positive and IDO1(−) negative cases. Pure TCMR was observed in 27.6% of IDO1(+) positive cases and in 42.2% of IDO1(−) negativecases, with a *p* value of 0.10. This suggests that the presence or absence of IDO1 may not significantly influence the occurrence of TCMR as a standalone rejection type. Similarly, when examining pure antibody-mediated rejection (AMR), the difference in prevalence between IDO1(+) positive and IDO1(−) negative cases also failed to reach statistical significance. Pure AMR was found in 1 (1.3%) IDO1(+) positive case and in 2 (4.4%) IDO1(−) negative cases, yielding a *p* value of 0.28. These results suggest that IDO1 expression may not be a major determinant in the development of pure AMR episodes.

## 4. Discussion

The findings from our pioneering study have revealed intriguing insights into the role of indoleamine 2,3-dioxygenase 1 (IDO1) in kidney transplantation, specifically concerning its potential impact on immunological injuries and acute rejection episodes.

To highlight the significance of IDO1 expression, we have unraveled its potential protective influence on immunological transplant injury. Our comprehensive analysis of biopsy samples from a substantial cohort of kidney transplant recipients demonstrates a compelling association between IDO1 expression in tubules and rejection incidence, particularly in T-cell mediated rejection (TCMR) and antibody-mediated rejection (AMR). In addition, several studies have also shown promising results in this area [48,49,50,51].

A central finding from our investigation is the pronounced difference in rejection incidence between patients with and without IDO1 expression in tubules. Those lacking IDO1 demonstrated a significantly higher risk of rejection, with rejection rates reaching 62.2%, while IDO1(+) positive patients experienced lower rejection rates at 32.9%. This statistically significant difference highlights the clinical relevance of IDO1 as a potential biomarker for assessing rejection risk in kidney transplant recipients, and it corroborates our previous summary [13]. Our study’s impact is further emphasized by the distinct associations between IDO1 expression and specific rejection types. We observed that the lack of IDO1 was not only associated with a higher risk of AMR, with 20% of IDO1(−) negative patients experiencing AMR episodes compared to only 5.2% of IDO1(+) positive patients, but also correlated with a higher risk of TCMR, with rejection rates of 57.8% and 31.6% in IDO1(−) negative and IDO1(+) positive patients, respectively. These findings underscore the complex and multifaceted nature of IDO1’s immunoregulatory functions, which may vary depending on the specific rejection pathway. Another significant finding was revealed when comparing the interval between transplantation and biopsy in IDO1(−) negative and IDO1(+) positive groups, where, evidently, in the IDO1(+) positive group, more time had elapsed until the biopsy realization.

The potential protective effect of IDO1 against acute rejection, particularly in AMR and TCMR, unveils new opportunities for therapeutic strategies. Enhancing IDO1 expression or exploiting its immunomodulatory effects could lead to innovative interventions that dampen harmful immune responses and promote immune tolerance. Such targeted approaches may ultimately improve graft survival and patient outcomes, addressing the persistent challenge of rejection in kidney transplantation. Recent findings have revealed IDO1’s significance in multiple different areas, such as neurology [52].

While our study provides valuable insights, it is crucial to acknowledge certain limitations. The retrospective nature and sample size may introduce biases and confounding factors, warranting validation through larger, prospective studies to bolster the statistical significance of our findings. Additionally, future investigations should focus on unraveling the precise mechanisms through which IDO1 influences humoral injury and AMR, further illuminating its role in kidney transplantation.

Nevertheless, researchers have explored the potential of IDO1 as a biomarker for transplant rejection. Monitoring IDO1 levels in the serum of transplant recipients could provide insights into the immune status and the potential risk of rejection. Elevated IDO1 activity might suggest that the immune system is trying to establish tolerance and reduce the chances of rejection. Suarez et al. [53] have found serum IDO1 activity to be connected with a higher risk of acute rejection in patients after heart transplantation. In addition, Weng et al. [54] have determined that IDO1 expression in peripheral blood was associated with more extreme rejection of transplanted livers in rats. Finally, Halloran et al. [55] have found that IDO1 presence correlates with antibody-mediated rejection, which corresponds to donor-derived cell-free DNA, possibly another rejection marker.

IDO1’s immunosuppressive properties are primarily linked to its role in tryptophan metabolism [56,57]. Tryptophan is an essential amino acid required for T-cell proliferation and function [58]. By degrading tryptophan, IDO1 can create an environment that inhibits T-cell activation and proliferation, thus suppressing the immune response [59]. Moreover, the metabolites produced along the kynurenine pathway can have immunosuppressive effects on various immune cell populations [60,61].

In the context of transplanted organs, cells within the transplanted tissue can express IDO1 in response to immune activation [14]. This local expression of IDO1 can contribute to creating an immunosuppressive micro-environment within the transplanted organ. This immunosuppressive environment is believed to be beneficial for promoting graft tolerance and reducing the risk of rejection [29,62].

Given IDO1’s role in immune regulation and its potential as both a marker for rejection and an immunosuppressive factor, researchers have explored IDO1-targeted therapies for improving cancer outcomes [63,64,65,66,67,68]. Modulating IDO1 activity could be a strategy to promote immune tolerance and reduce the need for aggressive immunosuppressive drugs, which can have significant side effects.

However, it is important to note that the role of IDO1 in transplantation is complex and context-dependent. While IDO1 can contribute to immune tolerance, its over-activation might also lead to immune escape from certain pathogens or tumors [69,70]. Therefore, a delicate balance between immune suppression and maintaining the ability to fight off threats needs to be maintained.

It has to be emphasized that IDO1’s role in transplantation involves its function as both a potential serum biomarker for rejection risk and an immunosuppressive factor in transplant cells. Its manipulation has the potential to improve transplant outcomes, but thorough research is needed to fully understand the intricacies of its effects and interactions within the immune system.

The pronounced disparity in the duration between transplantation and biopsy, notably observed in the IDO1(+) positive group with a median period of 203 weeks, hints at a potential protective role of IDO1 in kidney transplants. This extended time frame may signify a unique immunomodulatory function of IDO1, possibly contributing to a more stable and resilient post-transplant environment. The prolonged period before biopsy in IDO1(+) positive individuals suggests that the expression of IDO1 could be associated with a dampening of immune responses or the mitigation of alloreactivity, fostering a conducive milieu for graft tolerance.

### 4.1. Limitations of Immunohistochemistry for IDO1 Detection

#### 4.1.1. Technical Limitations

Immunohistochemistry (IHC), while widely utilized, comes with inherent technical limitations. Variability in tissue fixation, antigen retrieval methods, and antibody specificity can contribute to variations in staining intensity and pattern. In our study, these technical factors may have influenced the interpretation of IDO1 expression, potentially affecting the sensitivity and specificity of our findings.

#### 4.1.2. Qualitative Nature of IHC

Immunohistochemistry provides qualitative rather than quantitative data. The designation of biopsies as IDO1(+) positivee based on any degree of staining introduces subjectivity. This qualitative approach might not capture subtle variations in IDO1 expression levels, potentially impacting the accuracy of associations with transplant rejection.

#### 4.1.3. Timing and Dynamics of IDO1 Expression

The dynamics of IDO1 expression in renal tubules remain incompletely understood. The temporal aspects of IDO1 induction and its correlation with rejection events are complex and may not be fully captured by a single biopsy. This temporal variation could introduce uncertainties into the sensitivity and specificity of the observed link between IDO1 and rejection.

#### 4.1.4. Confounding Factors

The designation of biopsies as IDO1(+) positive, irrespective of staining intensity, may introduce confounding factors. The presence of IDO1 alone does not necessarily elucidate the underlying mechanisms or the functional implications of its expression. Factors influencing IDO1 expression, such as inflammation or other local tissue conditions, could potentially confound the association with rejection.

#### 4.1.5. Unknowns and Future Directions

Importantly, our study underscores the existing gaps in understanding when and why IDO1 is present in renal tubules. Addressing these unknowns is crucial for refining the specificity and sensitivity of IDO1 as a biomarker for rejection. Future research should aim to elucidate the temporal dynamics of IDO1 expression and its functional implications in the context of transplant rejection.

In summary, our pioneering study has shed light on the role of IDO1 as a potential indicator and therapeutic target in kidney transplantation. The association between IDO1 expression and rejection incidence, particularly in TCMR and AMR, underscores its significance in assessing immunological injuries. This discovery offers promising prospects for personalized interventions, paving the way for enhanced graft outcomes and improved long-term success in kidney transplantation. Despite its limitations, the study reveals an exciting avenue for future research in the field of transplantation medicine.

## 5. Conclusions

Ultimately, our research suggests an association between IDO1 expression in kidney transplant tubules and a reduced incidence of rejection, indicating a potential protective role. Considering the correlation with favorable transplant outcomes, evaluating IDO1 expression in renal transplant biopsies could enhance immunological risk assessment; however, validation in a larger patient cohort is needed.

Our study opens avenues for kidney transplantation research and interventions, proposing IDO1 as a target for therapies aiming to bolster its expression or exploit its immunomodulatory effects. The use of IDO1 as a predictive biomarker may facilitate early identification of patients at higher risk of rejection, allowing for timely interventions and improved graft outcomes.

In conclusion, our insights into IDO1’s involvement in kidney transplants, especially in antibody-mediated rejection, contribute to ongoing efforts to ensure transplant success. As we delve deeper into IDO1’s role, its potential applications may extend beyond kidney transplantation, offering broader insights into graft rejection mechanisms and immune regulation. The identification of IDO1 as a marker for rejection risk assessment holds promise for personalized immunosuppressive strategies, potentially improving renal transplant survival and informing broader organ transplantation practices.

## Figures and Tables

**Table 1:** Characteristics of patients according to the expression of indoleamine 2,3-dioxygenase (IDO1) in tubules. Data are presented as the mean ± standard deviation.

Patient Characteristics	IDO1 Expression in Tubules IDO1(+) Positive n = 76	IDO1 Expression in Tubules IDO1(−) Negative n = 45	*p*
Recipient’s age (years)	39.8 ± 14	45.6 ± 14	0.03
Male gender (n, %)	54 (71%)	31 (68.8%)	0.8
Number of HLA * ABDR ** mismatches	3.52 ± 0.9	3.57 ± 1.3	0.84
A	1.32 ± 0.5	1.27 ± 0.5	0.71
B	1.48 ± 0.5	1.27 ± 0.6	0.08
DR	0.71 ± 0.5	1.027 ± 0.5	0.007
Percentage of pre-sensitized patients			
PRA *** < 10%	37/49 (75.5%)	19/29 (65.5%)	0.34
PRA 10–50%	10/49 (23.1%)	7/29 (13.5%)	0.69
PRA > 50%	2/49 (4%)	3/29 (10%)	0.25
Cold ischemia time (hours)	22.2 ± 8.1	21.9 ± 8.6	0.87
Donor male gender (n, %)	36/59 (61%)	17/30 (56%)	0.69
Donor age (years)	46.8 ± 13.7	50.5 ± 17.3	0.34

* Human Leukocyte Antigen. ** HLA-A, HLA-B (Class I) and HLA-DR (Class II)—specific classes of HLA genes. *** Panel Reactive Antibody.

**Table 2:** Types of initial immunosuppression used after kidney transplantation in the group of patients, relating to the expression of indoleamine 2,3-dioxygenase (IDO1) in tubules. Specific quantities and percentages of positive and negative cases concerning a particular factor are presented.

Initial Immunosuppression	IDO1 Expression in Tubules IDO1(+) Positive n = 76	IDO1 Expression in Tubules IDO1(−) Negative n = 45	*p*
Tacrolimus	53 (70%)	32 (71%)	0.87
Cyclosporin	23 (30%)	13 (29%)	0.81
MMF/MPA *	76 (100%)	45 (100%)	NS ***
Azathioprine	2 (2.6%)	0 (0%)	NS
Anti-CD25 ** therapy	1 (1.3%)	1 (2.2%)	0.7

* Mycophenolate Mofetil or Mycophenolic Acid. ** Interleukin-2 receptor alpha chain. *** Not significant.

**Table 3:** Causes of chronic renal failure in patients, relating to the expression of indoleamine 2,3-dioxygenase (IDO1) in tubules. Specific quantities and percentages of positive and negative cases concerning a particular factor are presented.

Cause of Chronic Renal Failure	IDO1 Expression in Tubules IDO1(+) Positive n = 76	IDO1 Expression in Tubules IDO1(−) Negative n = 45	*p*
Chronic glomerulonephritis	33 (43. 4%)	18 (40%)	0.66
Diabetic nephropathy	16 (21.3%)	8 (17.8%)	0.66
Hypertonic nephropathy	6 (8%)	5 (11.1%)	0.71
Polycystic kidney disease	8 (10.7%)	3 (6.67%)	0.47
Pyelonephritis	3 (4%)	3 (6.67%)	0.51
Other	10 (13.3%)	8 (17.8%)	0.49

**Table 4:** Summary of the results and types of rejection related to the expression of indoleamine 2,3-dioxygenase (IDO1). Specific quantities and percentages of positive and negative cases concerning a particular factor are presented.

Biopsy Diagnosis	IDO1 Expression in Tubules IDO1(+) Positive n = 76	IDO1 Expression in Tubules IDO1(−) Negative n = 45	*p*
Rejections (all)	25/76 (32.9%)	28/45 (62.2%)	0.0017
AMR * (including pure and mixed AMR)	4 (5.2%)	9 (20%)	0.0085
TCMR ** (including pure and mixed TCMR)	24 (31.6%)	26 (57.8%)	0.0046
AMR (pure)	1 (1.3%)	2 (4.4%)	0.28
TCMR (pure)	21 (27.69%)	19 (42.2%)	0.10

* Antibody-mediated rejection. ** T-cell-mediated rejection.

## Data Availability

The data presented in this study are available on request from the corresponding authors. The data are not publicly available due to privacy policy.

## Abbreviations

The following abbreviations are used in this manuscript:

IDO1	indoleamine 2,3-dioxygenase 1
TE	tubular epithelium
HLA antibody	human leukocyte antigen antibody
non-HLA antibody	non-human leukocyte antigen antibody
PRA	panel reactive antibody
AMR	antibody-mediated rejection
TCMR	T-cell-mediated rejection
MMF	mycophenolate mofetil
MPA	mycophenolic acid
HE	hematoxylin and eosin staining
PAS	periodic acid–Schiff staining
IHC	immunohistochemistry
